# The Alteration of L-Carnitine Transport and Pretreatment Effect under Glutamate Cytotoxicity on Motor Neuron-Like NSC-34 Lines

**DOI:** 10.3390/pharmaceutics13040551

**Published:** 2021-04-14

**Authors:** Asmita Gyawali, Seung Jae Hyeon, Hoon Ryu, Young-Sook Kang

**Affiliations:** 1 College of Pharmacy and Drug Information Research Institute, Sookmyung Women’s University, Seoul 04310, Korea; gyawaliashu@sookmyung.ac.kr; 2 Laboratory for Brain Gene Regulation and Epigenetics, Brain Science Institute, Korea Institute of Science and Technology, Seoul 02792, Korea; t15321@kist.re.kr (S.J.H.); hoonryu@kist.re.kr (H.R.)

**Keywords:** L-carnitine, uptake study, OCTN1 and 2, NSC-34 cell lines, amyotrophic lateral sclerosis, glutamate neurotoxicity

## Abstract

L-Carnitine (LC) is essential for transporting fatty acids to the mitochondria for β-oxidation. This study was performed to examine the alteration of the LC transport system in wild type (WT, NSC-34/hSOD1^WT^) and mutant type (MT, NSC-34/hSOD1^G93A^) amyotrophic lateral sclerosis (ALS) models. The uptake of [^3^H]L-carnitine was dependent on time, temperature, concentration, sodium, pH, and energy in both cell lines. The Michaelis–Menten constant (K_m_) value as well as maximum transport velocity (V_max_) indicated that the MT cell lines showed the higher affinity and lower capacity transport system, compared to that of the WT cell lines. Additionally, LC uptake was inhibited by organic cationic compounds but unaffected by organic anions. OCTN1/slc22a4 and OCTN2/slc22a5 siRNA transfection study revealed both transporters are involved in LC transport in NSC-34 cell lines. Additionally, slc22a4 and slc22a5 was significantly decreased in mouse MT models compared with that in ALS WT littermate models in the immune-reactivity study. [^3^H]L-Carnitine uptake and mRNA expression pattern showed the pretreatment of LC and acetyl L-carnitine (ALC) attenuated glutamate induced neurotoxicity in NSC-34 cell lines. These findings indicate that LC and ALC supplementation can prevent the neurotoxicity and neuro-inflammation induced by glutamate in motor neurons.

## 1. Introduction

Amyotrophic lateral sclerosis (ALS), which is a devastating neurodegenerative disease, causes motor neuronal loss in the brain stem and spinal cord, resulting in paralysis, muscle wasting and respiratory failure, which ultimately leads to death. The demand for extending the quality of life in ALS patients is increasing, mainly due to the lack of an effective cure for this disease [1,2]. Familial ALS (FALS) is apparently caused by the mutation of a gene situated on chromosome 21q that encodes copper/zinc superoxide dismutase (Cu/Zn SOD, SOD1) [3,4]. The mutation leading to FALS causes pathological symptoms, such as glutamate excitotoxicity, oxidative stress, protein misfolding, mitochondrial dysfunction, abnormalities in the axonal and electron transport chains, and defective ATP synthesis [4,5,6]. Despite these findings, the etiology of motor neuronal dysfunction in ALS as well as the mechanisms underlying such dysfunction remain elusive. In transgenic animal ALS models, β-hydroxy-γ-trimethylammonium butyrate (carnitine) levels in the spinal cord were found to decrease with disease progression, and such carnitine deficiency reportedly enhanced the downregulation of a patient’s mental state [6,7].

Carnitine plays a major role in the transportation of long chain fatty acids to the mitochondria for β-oxidation, a process which generates ATP. Under physiological conditions, oxidization of fatty acids primarily involves mitochondrial β-oxidation, which is an important energy production mechanism found in mammals [8,9]. The mitochondrial membrane is impermeable to acyl-CoA, and therefore fatty acids are conjugated with carnitine in order to enter mitochondria and then converted to acyl-CoA [8]. When administered to human neuronal cells, the mitochondrial energy-metabolizing compound L-carnitine (LC) accumulates in the form of the free compound and its acyl derivatives, which increase mitochondrial functions. This signifies that it may play a preventative role under pathological conditions induced by motor neuron diseases, such as ALS [9]. Additionally, carnitine exhibits antioxidant properties and free radical scavenging effects and plays a preventive role against mitochondrial injury, by reducing free fatty acid long chains, which distort the mitochondrial membrane. This, in turn, increases the survival rate in mouse neurodegenerative disease models by ameliorating motor neuronal activity [6,10]. In addition, LC exerts anti-inflammatory effects on traumatic brain injuries and glutamate excitotoxicity, and thereby exerts a neuroprotective effect on neurodegenerative diseases. Thus, investigating the LC transportation process may be considered mandatory for future studies [11,12].

Organic cationic/carnitine transporters (OCTN) are unique zwitter-ionic transporters expressed in mice. These are categorized under the solute carrier family (SLC) and its members 4, 5 and 21 are represented by OCTN1/Slc22a4, OCTN2/Slc22a5, and OCTN3/Slc22a21 [12,13]. Apparently, ubiquitous expression of OCTN1 and the other 2 transporters is associated with the transportation of anti-oxidant compounds, such as ergothioneine and LC, in a sodium dependent manner, and therefore, tissues that are unable to produce carnitine receive carnitine via these transporters [13,14]. Furthermore, fatty acids are unable to cross the inner membrane of mitochondria in tissues deficient in carnitine, leading to mitochondrial disorders. However, pretreatment with carnitine can be utilized to maintain carnitine homeostasis, which facilitates the translocation of fatty acyl units required for β-oxidation to the mitochondrial matrix. This mechanism prevents carnitine deficiency-related mitochondrial disorders [9,12].

Previous studies have demonstrated that concentrations of various compounds, such as amino acids and monocarboxylates, are altered in the mutant ALS model. Therefore, investigating the transportation and supplementation of LC is important for acquiring a better understanding of mammalian mitochondrial energy metabolism and the prevention of neurodegenerative diseases, such as ALS [15,16]. However, despite tremendous progress in related research, the causes of ALS remain largely unknown and finding a permanent preventive solution to ALS remain problematic. In order to minimize the pathological features of FALS, transgenic mice overexpressing the human SOD1^G93A^ mutation were used to examine the neuroprotective effects of LC and ALC. In this study, we hypothesized that carnitine and ALC pretreatment may provide neuroprotective effects against glutamate-induced excitotoxicity in ALS model cell lines. Similarly, we investigated the effects of altering carnitine transportation, as well as carnitine pretreatment, on [^3^H]L-carnitine uptake in motor neurons, such as NSC-34 cell lines.

## 2. Materials and Methods

### 2.1. Materials

L-Carnitine hydrochloride [methyl-^3^H] (77.0 mCi/mmol) was purchased from Perkin-Elmer (Waltham, MA, USA). All chemicals and reagents were high-grade commercial products purchased from Sigma-Aldrich (Merck group; St. Louis, MO, USA).

### 2.2. Cell Culture

Motor neuron-like (NSC-34) cell lines, were prepared by transfecting it with pClneo expression vector containing wild type (WT/NSC-34/hSOD1^WT^) and mutant SOD1^G93A^ (MT/NSC-34/hSOD1^G93A^) [15,16,17]. WT cells of passage number #5–18 and MT cells of passage number #5–20, cultured and subculture were done and seeded on the collagen coated type-I dish with Dulbecco’s modified Eagle’s medium (Invitrogen, San Diego, CA, USA), supplemented with 100 U/mL penicillin, 100 μg/mL streptomycin (Invitrogen), and 10% fetal bovine serum at 37 °C in a humidified atmosphere under 5% CO_2_ according to previously described methods [15,16,17]. For the experiment, cells were sub-cultured and seeded on rat tail collagen type I-coated 24-well culture plates (IWAKI, Tokyo, Japan) at a density of 1 × 10^5^ cells/well, and 6 well plates at a density of 1 × 10^5^ cells/well. Cultured cells were incubated for two days at 37 °C, until the cultures reached confluence and then used to study uptake, mRNA expression levels and cell viability, among others.

### 2.3. Uptake Study on NSC-34 Cell Lines

[^3^H]L-Carnitine uptake in NSC-34 cells was studied based on a previously described method [17]. Cells were washed thrice at 37 °C using 1 mL of extracellular fluid (ECF) buffer, which was prepared according to a previous method. A [^3^H]L-carnitine (32.5 nM) labeled compound was then added in the presence or absence of inhibitors for a designated time period at 37 °C [16]. Uptake was terminated by washing the cells thrice with ice cold (4 °C) ECF buffer [18], following which the cells were solubilized with 1 N NaOH + PBS, and radioactivity of the compound was measured using a liquid scintillation counter (LSC LS6500; Beckman, Fullerton, CA, USA). The results were plotted using the following equation [17]:

(1) $$\mathrm{Cell}\;\mathrm{to}\;\mathrm{medium}\;\mathrm{ratio}=\frac{\left[14C\right]\mathrm{dpm}\;\mathrm{per}\;\mathrm{cell}\;\mathrm{protein}\;\left(\mathrm{mg}\right)}{\left[14C\right]\mathrm{dpm}\;\mathrm{per}\;\mathrm{uL}\;\mathrm{medium}}$$

To analyze sodium dependency, NaCl and NaHCO_3_ in the ECF buffer was replaced with chloride and N-methyl-D-glucamine (NMDG), respectively. Similarly, to evaluate energy dependency, 20 min pretreatment was performed using 0.1% sodium azide (NaN_3_), 25 mM rotenone (0.2% DMSO), and 10 mM of 3-O-methyl glucose (which replaced 10 mM D-glucose in the uptake buffer to reduce metabolic energy). Similarly, pH in the ECF buffer was adjusted to 6.0, 7.4, and 8.4 using HCl or NaOH to test for pH dependency according to a previously described experimental procedure [17,19].

### 2.4. Kinetic Analysis

For kinetic studies [17], the Michaelis-Menten constant (K_m_), maximum uptake rate (V_max_), and the first-order constant for the non-saturable component (K_d_) were estimated for NSC-34 cell ‘via Equation (2) using a nonlinear least-squares regression analysis program (Sigma Plot version 12, Systat Software Inc., Richmond, CA, USA).

V = [V_max1_·C/(K_m1_+C) + V_max2_·C/(K_m2_+C)] + K_d_·C (2)

where, V and C are the initial uptake rate of [^3^H]-L-carnitine at 15 min and the concentration of unlabeled carnitine, respectively.

For the competitive inhibition study [17,20], low-affinity L-carnitine concentrations were used in the presence of quinidine, pyrilamine, diphenhydramine and metformin at a concentration of 0.5 mM, and inhibition constant (K_i_) was calculated using Equation (3).

V = V_max_·C/[K_m_ (1+I/K_i_) + C] + K_d_·C (3)

where, I represents the concentration of inhibitors.

### 2.5. OCTN1 and 2 Small Interfering RNA (siRNA) Transfection Study

For gene knockdown, forward primer sequence: CGTGACAGAGTGGAATCTGGT, reverse primer sequence: GAGAACGCCTACGAAGAACAG for mOCTN1/slc22a4, forward primer sequence: ACTGTGCCAGGGGTGCTAT, reverse primer sequence: TCCGTGTTCGGATCAGATCATAA for mOCTN2/slc22a5 and non-targeting pool (negative control) siRNAs ((GE Healthcare Dharmacon, Inc., Lafayette, CO, USA), were transfected into NSC-34 WT and MT cells for 24 h, at a concentration of 200 nM, using Lipofectamine^®^ 2000 (Invitrogen). After the designated time interval, mRNA expression level and [^3^H]L-carnitine uptake in siRNA-transfected NSC-34 cell lines were evaluated [21].

### 2.6. Quantitative Real Time-Polymerase Chain Reaction (qRT-PCR) Study

Total RNA was extracted from NSC-34 WT and MT cell lines, using RNeasy kit (Qiagen, Valencia, CA, USA) according to the manufacturer’s instructions. Single-stranded cDNA was prepared from total RNA using a high-capacity RNA-to-cDNA kit. The StepOnePlus real-time PCR system (AB Applied Biosystems, Foster City, CA, USA), in combination with TaqMan^®^ Gene Expression Master Mix (Applied Biosystems), was used according to the manufacturer’s protocol for quantitative real-time PCR (qPCR) analysis. In addition, mOCTN1/slc22a4 and mOCTN2/slc22a5 expression levels were normalized to the housekeeping gene, GAPDH using a gene amplification system (MyCycler; BioRad Laboratories Inc., Hercules, CA, USA).

### 2.7. ALS Mouse Model

Male transgenic ALS mice of the mt-SOD1 (G93A) H1 high-expresser strain (Jackson Laboratories, Bar Harbor, ME, USA) were bred with females with a similar background (B6/SJLF1). This study used postmortem spinal cord tissue sections that were generously provided from Dr. Kowall and Dr. Lee in VA Boston Healthcare System (IACUC approval. #311-J-102510, 24 October 2013)

### 2.8. Histopathology and Bright Field Microscopy

In order to determine changes of Slc22a4 and Slc22a5 immunoreactivity, WT littermate and SOD1 (G93A) mice at 120–150 days of age were perfused via using 4% buffered paraformaldehyde in PBS. Spinal cords were extracted and serially sectioned at 30 μm of thickness by cryostat. Lumbar spinal cord sections (2 sections per animal) were used for immunostaining to detect the level of Slc22a4 and Slc22a5. First, the tissue sections were incubated for 1.5 h in a blocking solution (0.3% Triton-X, 2% goat serum, and 2% donkey serum in 0.1 M PBS) and then further incubated with primary antibodies [Slc22a4 (1:200 dilution) (Cat. No.: H00006583-A01, NOVUS) and Slc22a5 (1:200 dilution) (Cat. No.: ab180757, Abcam, Cambridge, MA, USA,)] in a blocking solution at 4 °C for 24 h. After washing three times with PBS, the tissue slides were processed with Vector ABC Kit (Vector Laboratories, Inc., Burlingame, CA, USA). The Slc22a4 and Slc22a5 signals were developed with DAB chromogen (Thermo Fisher Scientific, Meridian, Rockford, IL, USA). Images were then analyzed via using an Olympus microscope system (Olympus, Tokyo, Japan). The semi-quantification of immunoreactivity was analyzed by NIH ImageJ. Especially, the intensity of Slc22a4 and Slc22a5 signals was counted from 5~10 motor neurons per section.

### 2.9. Glutamate Neurotoxicity in NSC-34 Cell Lines

NSC-34 cell lines were exposed to glutamate with or without L-carnitine (LC) and acetyl L-carnitine (ALC) at a 1 mM concentration for 24 h. To assess the cell survival rate in ALS model cell lines, an MTT [3-(4,5-dimethyldiazol-2-yl)-2,5-diphenyltetrazoliumbromide] assay was performed, following which [^3^H]L-carnitine uptake, and slc22a4 mRNA expression level were examined [22]. Cell lines were exposed to LC and ALC for 15 min, following which glutamate was added and incubated for 24 h. Next, MTT (5 mg/mL) solution was added to each well and stored at 37 °C in the dark for 3 h. After the solution turned purple due to precipitation of formazan, DMSO was added and absorbance was evaluated using an Infinite F200 PRO microplate reader (Tecan Trading AG, Männedorf, Switzerland) at a wavelength of 550 nM, as previously described [22]. Here, glutamate was used to induce glutamate-induced neurotoxicity, and LC and ALC were added to examine their neuroprotective effects on both the NSC-34 cell lines [17].

### 2.10. Data Analysis

Statistical analyses were conducted via the unpaired 2-tailed Student’s t-test or one-way ANOVA followed by Dunnett’s post-hoc test. Sigma Plot (version 12, Systat Software Inc., Richmond, CA, USA) was used to create figures.

## 3. Results

### 3.1. The Characteristics of [^3^H]L-Carnitine Transport in NSC-34 Cell Lines

**Figure 1:**
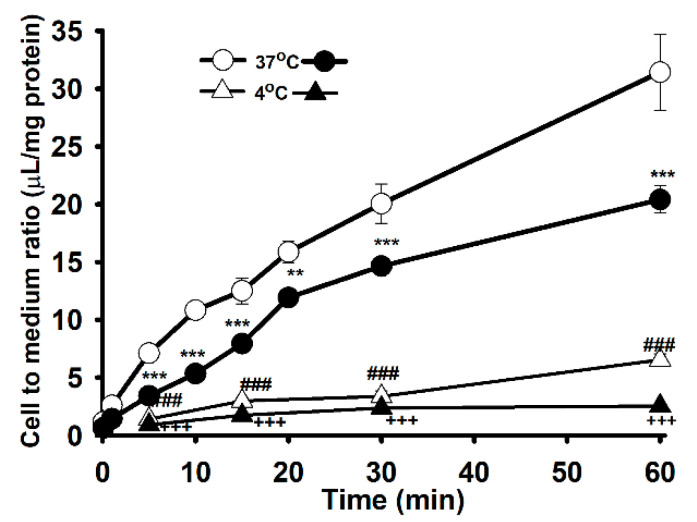
Time and temperature dependent uptake of [^3^H]L-carnitine (32.5 nM) in NSC-34 cell lines. The uptake was monitored from 1 to 60 min at 37 °C and 4 °C under physiological pH. Open circles (ο) and triangle (Δ) represent the wild type (WT, NSC-34hSOD1^wt^), and closed circles (●) and triangle (▲) denotes the mutant type (MT, NSC-34 hSOD1^G93A^). Each point represents the mean ± S.E.M (*n* = 3–4). *** *p* < 0.001, ** *p* < 0.01 and ### *p* < 0.001 indicate significant differences from the WT 37 °C, and +++ *p* < 0.001 represents significant difference from MT 37 °C.

**Figure 2:**
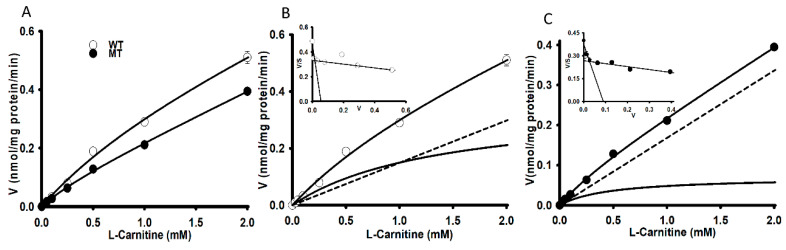
Concentration-dependent uptake of [^3^H]L-carnitine in NSC-34 cell lines (**A**). [^3^H]L-Carnitine uptake was carried out using a unlabeled L-carnitine concentration of 0–2 mM in NSC-34 WT (**B**) and MT (**C**) cell lines at 37 °C for 15 min under physiological pH. Each point represents the mean ± S.E.M (*n* = 3).

Carnitine uptake was studied in order to examine the effects of the [^3^H]L-carnitine transport system on WT and MT NSC-34 cell lines. The uptake of [^3^H]L-carnitine was time-and temperature-dependent until 60 min in both NSC-34 WT and MT cell lines (Figure 1). The uptake of carnitine was found significantly decreased in the MT cell lines compared to the WT cell lines at both 37 °C and 4 °C. Uptake in both cell lines was diminished under an ice-cold temperature of 4 °C (Figure 1). The uptake was linear until 15 min, following which the uptake rate in the MT cell line was lowered compared to that in the WT cell lines. Thus, the uptake time for further experiment was set to 15 min. Next, 0–2 mM unlabeled L-carnitine was used to examine the kinetic parameters of the [^3^H]L-carnitine transporter in NSC-34 cell lines (Figure 2). The results indicated that the Michaelis-Menten constant for high-affinity sites (K_m1_) and the maximum uptake rate (V_max1_) were 1.94 ± 0.34 µM and 0.295 ± 0.059 pmol/mg protein/min, respectively, for the WT cell line (Figure 2B), and 1.96 ± 0.27 µM and 0.191 ± 0.032 pmol/mg protein/min, respectively, for the MT cell line (Figure 2C). Similarly, for low affinity sites, K_m2_ and V_max2_ were 994 ± 34 µM and 259 ± 9 pmol/mg protein/min, respectively, for WT and 374 ± 89 µM and 62.3 ± 13.3 pmol/mg protein/min, respectively, for MT (Figure 2 and Table 1). The non-saturable transport clearance (K_d_) values for WT and MT cell lines, were 0.149 (µL/(min/mg protein)) and 0.168 (µL/(min/mg protein)), respectively (Table 1). These results demonstrated that high affinity and low capacity carnitine transport system are present in both the NSC-34 cell lines at high affinity sites but at low affinity sites, MT showed the higher affinity and lower capacity transport system, which showed the significant different as compared to that of the WT cell lines. In addition, the embedded graph associated with the Eadie-Scatchard plot showed two straight lines, indicating that two saturable processes were involved in L-carnitine transport in both WT and MT cell lines (Figure 2B,C). These results indicated that the carnitine transport system in NSC-34 cell lines was a time-, temperature- and concentration-dependent and carrier-mediated transport system.

**Figure 3:**
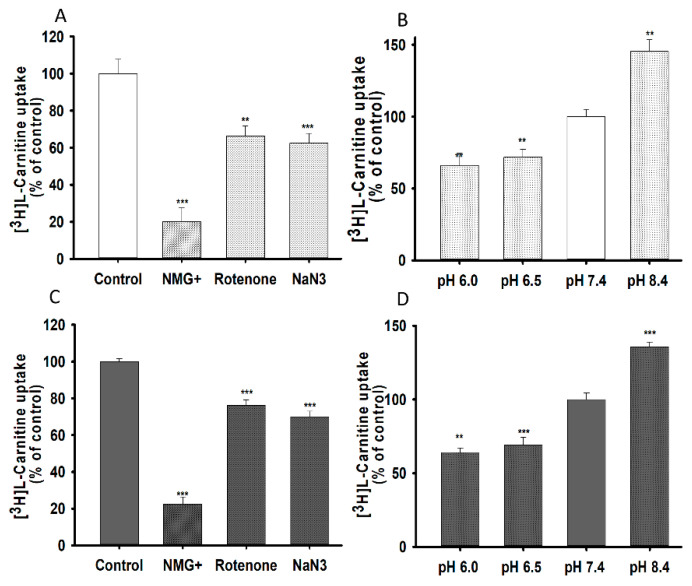
Sodium, energy and pH dependent uptake of [^3^H]L-carnitine in NSC-34 cell lines in 15 min at 37 °C. Sodium azide (0.1%) and rotenone (25 µM) pretreatment was carried out for 20 min to deplete ATP. To test for sodium dependency, Na^+^ in transport buffer was replaced by NMG+ and pH dependency was tested for pH ranging from 6.0 to 8.4 and uptake was conducted in WT (**A**,**C**) and MT (**B**,**D**) cell lines. Each data represents the mean ± S.E.M (*n* = 4). *** *p* < 0.001 and ** *p* < 0.01 indicate significant differences from the control.

To examine the effect of ions and energy on the uptake of L-carnitine, sodium was replaced with N-methyl-D-glucamine, and a 20 min pretreatment with sodium azide (NaN_3_), and rotenone was used to deplete ATP (Figure 3A,B). Lower concentration of NaN_3_ and rotenone were not toxic for NSC-34 cell lines. Carnitine uptake was significantly decreased in the absence of ions and energy, indicating that such uptake was sodium-and energy-dependent in both NSC-34 cell lines (Figure 3A,B). Moreover, to examine the effect of extracellular pH on carnitine uptake, we tested the effect of acidic (6.0, 6.5) basic (8.4) and physiological (7.4) pH on 15 min of uptake at 37 °C in both NSC-34 cell lines. Carnitine uptake in both cell lines was markedly decreased at acidic pH, but significantly increased at basic pH, compared to that at physiological pH (Figure 3C,D). These results suggested that carnitine uptake in NSC-34 cell lines was sodium-, energy- and pH-dependent.

### 3.2. Structural Inhibition Analog Study in the Uptake of [^3^H]L-Carnitine by NSC-34 Cell Lines

To examine the transporter(s) responsible for transporting carnitine into NSC-34 cell lines (ALS model cell lines), cellular uptake of [^3^H]L-carnitine was monitored in the presence or absence of several transporter inhibitors at concentrations of 0.5 to 1 mM and a pH of 7.4, for 15 min. Carnitine uptake in both WT and MT cell lines was markedly inhibited in the presence of organic cation/carnitine transporter inhibitors, such as palmitoyl L-carnitine (PLC), γ-butyrobetaine (GBB), acetyl L-carnitine (ALC) and tetraethyl ammonium (TEA) (Table 2). Similarly, novel organic cationic transporter inhibitors, such as diphenhydramine (DPH), quinidine, pyrilamine, and verapamil also significantly inhibited carnitine uptake. However, large neutral amino acid transporter inhibitors, such as GABA, gabapentin, histidine, and dopamine, choline like transporter inhibitor; choline and organic anion transporter (OAT) inhibitors, including para-aminohippuric acid (PAH) and estrone-3 sulfate, did not induce a significant difference in the uptake of carnitine in both NSC-34 cell lines at pH 7.4 (Table 2).

**Figure 4:**
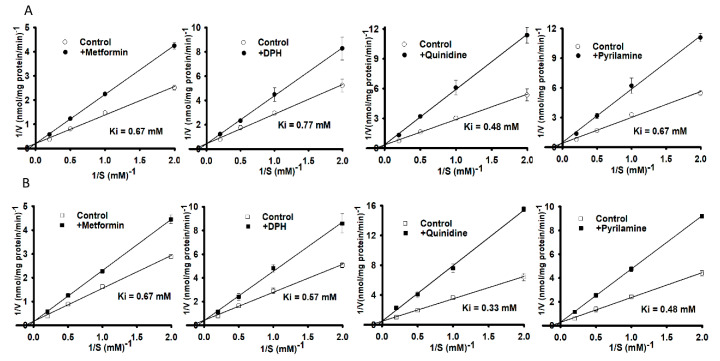
A kinetic inhibition study of [^3^H]L-carnitine in NSC-34 cell lines was performed via Lineweaver-Burk plots. Uptake for 15 min was evaluated in the presence or absence of 0.5 mM metformin, diphenhydramine (DPH), quinidine and pyrilamine in (**A**) WT (circle) and (**B**) MT (square) cell lines at 37 °C under physiological pH. Each value represents the mean ± S.E.M (*n* = 3) of three independent experiments.

After investigating the effect of transporter inhibitors, we examined kinetic inhibition in NSC-34 cell lines in the presence or absence of organic cationic compounds at a concentration of 0.5 mM for 15 min of carnitine uptake at a pH of 7.4. Lineweaver-Burk plots of [^3^H]L-carnitine uptake intersected at the ordinate axes, showing competitive inhibition in the presence or absence of metformin, DPH, quinidine and pyrilamine in both WT (Figure 4A) and MT (Figure 4B) NSC-34 cell lines. The K_i_ value corresponding to MT was slightly lower than that for the WT cell lines in this competitive inhibition study (Figure 4).

### 3.3. SiRNA Transfection and Carnitine Transporter Alteration Study in NSC-34 Cell Lines

**Figure 5:**
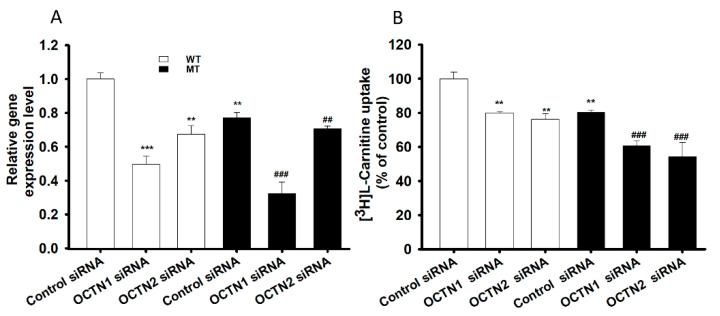
SiRNA transfection study in NSC-34 cell lines. Control, mOCTN1 and mOCTN2 siRNAs, at a concentration of 200 nM, were transfected using Lipofectamine 2000, and cell lines were examined for (**A**) mRNA expression levels using qRT-PCR and (**B**) [^3^H]L-carnitine uptake for 15 min at pH 7.4. Open space represents WT cell lines and dark space represents MT cell lines in both the figures. Each value represents the mean ± S.E.M (*n* = 4) of four independent experiments. *** *p* < 0.001 and ** *p* < 0.01 denote significant difference from the WT control, ### *p* < 0.001 and ## *p* < 0.01 denote significant difference from the respective MT control.

In order to examine the role of the main transporters of LC, NSC-34 cell lines were transfected using mOCTN1 (slc22a4), mOCTN2 (slc22a5) and control siRNA (negative control) then using the transfected cells, the mRNA expression levels and L-carnitine uptake was evaluated (Figure 5). mOCTN1 and mOCTN2 siRNA-transfected NSC-34 cell lines demonstrated a significant decrease in the slc22a4 and slc22a5 gene expression level, when normalized to the housekeeping gene (GAPDH) (Figure 5A), and the [3H]L-carnitine uptake rate compared to the negative control (control siRNA) (Figure 5B). Normal uptake results uniformly indicated that both gene expression and uptake rate were significantly decreased in MT cell lines compared with that in the WT cell lines (Figure 1, Figure 2 and Figure 5).

**Figure 6:**
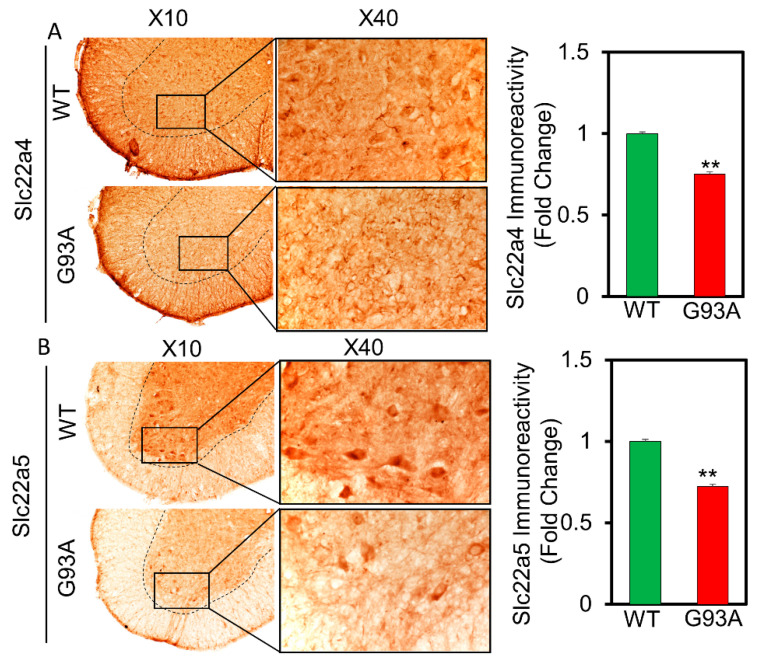
Carnitine transporters SLC22a4 (**A**) and SLC22a5 (**B**) immunoreactivity is decreased in motor neurons of the gray matter of spinal cord of ALS (G93A) mice (*n* = 5) in comparison to WT littermate mice (*n* = 5). Scale bar left: 100 µm, right: 40 µm. ** *p* < 0.01 significant difference from the WT mice.

**Figure A1:**
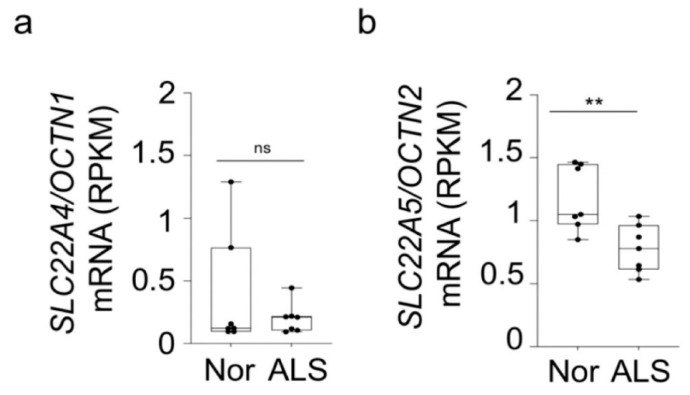
Motor neuronal expression of carnitine transporter is altered in the spinal cord of human ALS patients. (**a**) SLC22A4 is decreased in motor neurons of the spinal cord of human ALS patients (*n* = 7) compared to normal subjects (Nor) (*n* = 7), but it is not significant. (**b**) SLC22A5 is significantly decreased in motor neurons of the spinal cord of human ALS patients (*n* = 7) compared to normal subjects (Nor) (*n* = 7). ** Significantly different at *p* < 0.01. This data was retrieved and analyzed from previously publishes data (GSE76220_ALS_LCM_MPR_gene_expression [23]. Briefly, laser capture microdissection (LCM) method was used to collect motor neuron-specific RNA and pooled motor neuronal RNA was undergone high-throughput RNA sequencing. The relative expression of transcript was presented as reads per kilobase of transcript per million mapped reads (RPKM).

Then, we determined whether the expression of carnitine transporters is altered in the spinal cord of ALS transgenic [SOD1(G93A)] mice. Interestingly, the immunoreactivity of Slc22a4 and Slc22a5 carnitine transporters were decreased in the spinal cord gray matter and motor neurons of SOD1(G93A) mice compared to the WT littermates (Figure 6A,B). Similarly, to verify whether the carnitine transporters are altered in the spinal cord of human ALS patients, we retrieved and analyzed previously published data from high-throughput RNA sequencing of motor neuron-specific RNA and pooled motor neuronal RNA in normal subjects and ALS patients that were obtained by laser capture microdissection (LCM) (GSE76220_ALS_LCM_MPR_gene_expression [23]. The relative expression of transcript was presented as reads per kilobase of transcript per million mapped reads (RPKM). Importantly, concurrent with our ALS (G93A) mouse data, this data showed that SLC22A4 is decreased in motor neurons of the spinal cord of human ALS patients (*n* = 7) compared to normal subjects (*n* = 7) (Figure A1a). SLC22A5 was significantly decreased in motor neurons of the spinal cord of human ALS patients (*n* = 7) compared to normal subjects (*n* = 7), indicating the altered expression of carnitine transporters in human ALS patients (Figure A1b). These immunoreactivity results were consistent with the in vitro results (Figure 5 and Figure 6).

### 3.4. Glutamate Cytotoxicity and Neuroprotective Effect on the NSC-34 Cell Lines

**Figure 7:**
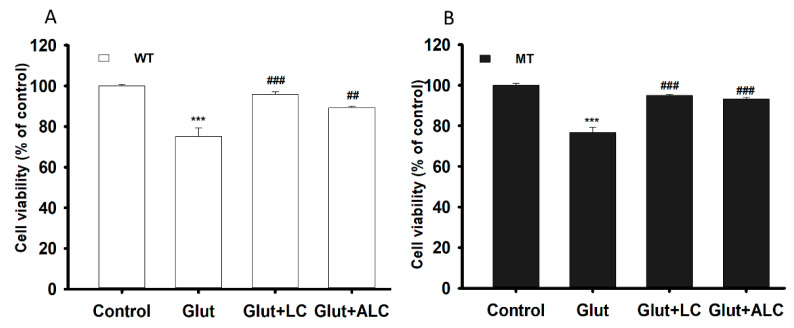
L-Carnitine (LC) and acetyl L-carnitine (ALC) ameliorates the neurotoxicity induced by the glutamate (Glut) in NSC-34 WT (**A**) and MT (**B**) cell lines. The cell viability was analyzed through the MTT assay after the pretreatment of glutamate with or without LC and ALC at a concentration of 1 mM for 24 h. Each value represents the mean ± S.E.M (*n* = 4). *** *p* < 0.001, versus control; ### *p* < 0.001 vs. glutamate; ## *p* < 0.01, vs. glutamate.

An MTT assay was performed on the ALS model of WT and MT cell lines following glutamate pretreatment to examine glutamate-induced cytotoxicity as well as the neuroprotective effect of LC and ALC under glutamate-induced cytotoxicity. Glutamate pre-incubation induced a significant decrease in cell viability compared to the control, but LC and ALC ameliorated glutamate-induced neuronal cell death in both NSC-34 cell lines (Figure 7A,B).

**Figure 8:**
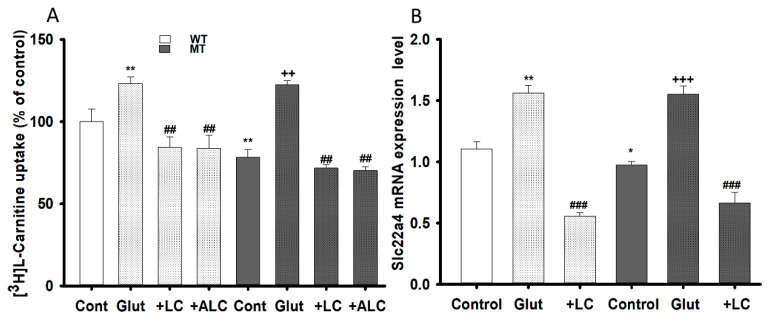
LC and ALC restoration effect following glutamate excito-toxicity on carnitine transport system. NSC-34 WT and MT cell lines were pretreated with Glutamate, LC and ALC (1 mM) for 24 h and [^3^H]L-carnitine uptake (**A**) and OCTN1/slc22a4 mRNA expression (**B**) were evaluated. Each value represents the mean ± S.E.M (*n* = 4); ** *p* < 0.01; * *p* < 0.05 were significantly different from the WT control, ### *p* < 0.001 and ## *p* < 0.01, significantly different from glutamate and +++ *p* < 0.001 and ++ *p* < 0.01, significantly different from MT control.

Similarly, the transport effects of LC and ALC pretreatment against glutamate excitotoxicity were examined by evaluating [^3^H]L-carnitine uptake and slc22a4 mRNA expression levels in both WT and MT cell lines. Pre-incubation with glutamate significantly increased the [^3^H]L-carnitine uptake rate, which was recovered of control level or decreased by pretreatment of LC and ALC in both NSC-34 cell lines (Figure 8A). The mRNA expression level of slc22a4 also showed results that were identical to that of uptake results (Figure 8B). An increase in both LC uptake and the slc22a4 mRNA expression level signified that glutamate pretreatment generated glutamate excitotoxicity and neurotoxicity in the NSC-34 cell lines, but that co-administering of LC and ALC prevented neuronal cell death (Figure 8).

## 4. Discussion

LC is an energy-metabolizing compound that facilitates mitochondrial transport of fatty acids, and any deficiency or undersupply of LC alters energy-metabolizing pathways, leading to fluctuations in ATP production. It also enhances mitochondrial dysfunction, and alters neuronal electric activity as well as calcium homeostasis, leading to the progression of ALS [5,24]. In this study, we investigated transport system of carnitine under physiological pH as well as its neuroprotective effects against glutamate cytotoxicity in motor neurons using NSC-34 WT and MT cell lines.

[^3^H]L-Carnitine uptake in NSC-34 cell lines was shown to be time-, temperature-, concentration-, ion-, energy-, and pH-dependent. The characteristics of its transport system were similar to those described in previous studies [25,26,27]. LC uptake results in NSC-34 cell lines were similar with the previously publish data which showed the sodium-, time- and temperature- dependency uptake by the substrates of OCTN1 and 2 (carnitine, ergothioneine, TEA and oxaliplatin) in the primary cultures of rat dorsal root ganglion neurons [28] (Figure 1). Time-and concentration-dependent uptake was depleted in the MT cell lines, indicating that it may be linked to a mutated or deleted transporter system as compared to WT cell lines (Figure 1, Figure 2); [16,17]. As mentioned previously, carnitine is an energy-metabolizing compound which shows energy-dependent uptake as well as transport characteristics that are similar to those of compounds transported by the OCTN1 transporter in neuronal cells because NaN_3_ and rotenone act by interfering with the electron transport chain in mitochondria and inhibition of the ATP synthase/production [14,26]. Kinetic parameters that were obtained suggested that two saturable processes were involved in the transport of carnitine in NSC-34 cell lines and that the transport system in MT (disease model) cell lines displayed a higher affinity and a lower capacity than that in the WT cell lines at low affinity sites (Figure 2, Table 1). Our results were substantiated by those of a previous study which demonstrated that a lower number of carnitine transporters were present in carnitine deficient JVS (disease) mice, which displayed decreased transport capacity [29]. In addition, our results showed that the high affinity transport system was present in disease model of neuronal cells, as described in a previous study [30]. Furthermore, [^3^H]L-carnitine uptake was strongly inhibited in the presence of organic cationic compounds, including inhibitors as well as the substrates of the OCTN1 and 2 transporters, such as PLC, GBB, ALC, quinidine, verapamil, metformin, pyrilamine, TEA, clonidine, and DPH, in both NSC-34 cell lines [31,32,33,34]. The LC uptake rate in the presence of OCTN inhibitors like PLC, GBB and DPH inhibited by around 63–78% in the WT cell lines but in the MT type expressed only 45–50% inhibition which can be related to the mutation or deletion of the OCTN transport system in the MT cell lines. However, amino acids and organic anionic compounds did not cause any significant differences in carnitine uptake by NSC-34 cell lines (Table 2) [25,30,35]. Next, the kinetic inhibition study demonstrated that LC uptake was competitively inhibited in the presence of organic cationic compounds (Figure 4). These results suggest that LC functions as a substrate that binds to both OCTN1 and 2, and that both OCTN1 and 2 competitively bind to LC, thereby determining the sensitivity of the LC transporter in NSC-34 cell lines [28].

Amino acids, neurotransmitters and monocarboxylic acids as well as many other low-molecular-weight polar compounds can be transported to various cell lines using multiple transport systems that enable these substances to cross plasma membranes [17,36]. Similarly, both OCTN1 and 2 are transporters that are involved in carnitine transport in neuron-like cell lines, such as NSC-34 WT and MT, wherein the transport characteristics of OCTN1 and 2, including sodium dependence and energy dependence, correspond to those of LC transport in NSC-34 cell lines [30,31] (Figure 5). Although OCTN3 is expressed in neuronal cell lines, its transport mechanism is independent of sodium, and it is not involved in LC transport in the NSC-34 cell lines [31,33,34]. The immunoreactivity study revealed the presence of both OCTN1 (slc22a4) and OCTN2 (slc22a5) transporters in the motor neurons of ALS mice. Our previous study, Slc7a5/LAT1 (large neutral amino acid transporter) expression pattern and recent study slc22a4 and slc22a5 expression pattern shown similar significant depletion in the ALS MT model mice compared to that of ALS WT littermates [16] (Figure 6). And the motor neuronal expression of SLC22A5 transporter is also found altered in the spinal cord of human ALS patients (Appendix A, Figure A1). Additionally, carnitine uptake and mRNA expression levels in both slc22a4 and slc22a5 siRNA knockdown cell lines were lower in the MT model than in those of the WT model; these results were consistent with those of the mouse ALS MT model (Figure 5, Figure 6). Lower transporters expression level is indicating the deletion or mutation of the transporter in the ALS cells in the MT mouse model [16,17,29].

On the other hand, glutamate is an important neurotransmitter, its pretreatment overstimulates its receptors, leading to the activation of glutamate receptors and an influx of Na^+^ and Cl^−^, which destabilizes intracellular Ca^2+^ homeostasis, thereby disrupting neuronal energy status and causing excitotoxic neuronal death, which constitutes the major root of neurodegenerative diseases, such as ALS [37]. Additionally, as mentioned by previous studies, glutamate-induced neuronal death mediated by the blockage of metabotropic glutamate receptors, activation of MAP-kinase, increased intracellular calcium influx and activation of nitric oxide synthase in the neuronal cells [38,39]. However, compounds containing trimethylamine groups, such as LC and ALC could prevent the glutamate induced cytotoxicity in the neuronal cell lines which might be due to enhancing the binding affinity of glutamate for the metabotropic glutamate receptors, inhibition of glutamate-induced activation of MAP-kinase and by depleting the calcium stores of the mitochondria and of the endoplasmic reticulum through phospholipase C activation [38,39]. In order to assess the neuroprotective effects of LC and ALC, pretreatment was performed in NSC-34 cell lines with or without addition of LC and ALC in glutamate (Figure 7). The effect of glutamate neurotoxicity increased both uptake and slc22a4 mRNA expression levels, but this effect was significantly reduced by the addition of LC and ALC in both NSC-34 cell lines (Figure 8). In a comparable study, taurine also showed a pattern similar to that shown by LC and ALC in the NSC-34 cell lines, indicating that these compounds exerted neuroprotective effects against glutamate, bilirubin and ammonia induced neurotoxicity in neuronal cells [39,40,41]. As stated in a previous report, LC and ALC are sufficient to prevent neuronal cell death, because the anti-oxidant and anti-inflammatory effects of LC and ALC improve mitochondrial function and protect against NMDA receptor toxicity by decreasing oxidative DNA damage and lipid peroxidation in neurons [5,11,42]. Similarly, pretreatment with LC attenuated neuronal apoptosis, oxidative stress and superoxide production, and increased carnitine palmitoyltransferase I and II, as well as protein levels and synaptic activity, thereby increasing the phosphorylation of protein kinase B (Akt) and the mechanistic target of rapamycin (mTOR) signaling pathway [11]. Previous in vivo studies have reported that, although LC levels in the diseased ALS model were decreased, supplementation of LC restored neuronal function by delaying disease onset and progression, and increased the life span of mouse SOD1^G93A^ mutant models [6]. These outcomes may play a vital role in the prevention of neurodegenerative diseases in the future.

Our results suggest that the energy-metabolizing compound carnitine can be transported to motor-neuron like cell lines using OCTN1 and OCTN2 transporters. LC transporter systems in the NSC-34 cell lines of mouse MT ALS models were altered compared to those of the WT control. Thus, LC and ALC show potential as good candidates for the prevention of neurodegenerative diseases, including ALS caused by the glutamate-induced motor neuronal cell death.

## Figures and Tables

**Table 1:** Kinetic analysis of [^3^H] L-carnitine uptake by NSC-34 cell lines.

Parameters	WT	MT
K_m1_ (µM)	1.94 ± 0.34	1.96 ± 0.27
K_m2_ (µM)	994 ± 34	374 ± 89 ***
V_max1_ (pmol/mg protein/min)	0.295 ± 0.059	0.191 ± 0.032
V_max2_ (pmol/mg protein/min)	259 ± 9	62.3 ± 13.3 ***
K_d_ (µL/(min/mg protein))	0.149	0.168

K_m_, V_max_, and K_d_ represent transport affinity, maximum transport velocity, and non-saturable transport clearance, respectively. *** *p* < 0.001, represent levels of significant difference from the respective WT K_m2_ and V_max2_.

**Table 2:** Effect of transporter inhibitors and substrates on [^3^H]L-carnitine uptake by NSC-34 cell lines.

	Concentration	[^3^H]Carnitine Uptake (% of Control)	
Compounds	(mM)	WT (hSOD1^WT^)	MT (hSOD1^G93A^)
Control	1	100 ± 6	100 ± 4
+Carnitine	1	60.6 ± 2.4 ***	56.4 ± 3.8 ***
+Palmitoyl-L-carnitine	0.5	36.7 ± 1.4 ***	51.2 ± 5.8 ***
+γ-Butyro betaine	0.5	37.3 ± 2.8 ***	55.3 ± 5.8 ***
+ALC	1	51.2 ± 7.5 ***	57.9 ± 4.8 ***
+Quinidine	1	44.7 ± 7.7 ***	40.0 ± 8.3 ***
+Verapamil	1	30.1 ± 8.7 ***	43.1 ± 7.9 ***
+Metformin	1	65.4 ± 9.3 **	51.8± 1.6 ***
+Pyrilamine	1	50.2 ± 7.7 ***	49.5 ± 8.2 ***
+TEA	1	45.9 ± 2.5 ***	50.0 ± 7.1 ***
+Clonidine	1	57.3 ± 3.8 ***	63.6 ± 1.7 ***
+Diphenhydramine	1	21.9 ± 2.2 ***	50.0 ± 5.2 ***
+Donepezil	0.5	29.7 ± 2.8 ***	46.7 ± 6.9 ***
+Nicotine	1	71.6 ± 8.7 **	69.9 ± 2.3 ***
+Paeonol	1	50.6 ± 2.8 ***	50.5 ± 1.2 ***
+Propranolol	1	72.8 ± 7.9 **	48.2 ± 1.5 ***
+MPP+	1	71.5 ± 2.4 ***	64.4 ± 9.1 ***
+Cimetidine	1	78.9 ± 5.7 **	73.2 ± 6.7 ***
+Choline	1	88.4 ± 7.3	88.3 ± 9.7
+Dopamine	1	90.4 ± 8.7	87.9 ± 6.3
+GABA	1	90.2 ± 9.6	91.4 ± 4.7
+Gabapentin	1	109 ± 8	101 ± 6
+Histidine	1	91.9 ± 6.6	110 ± 8
+Probenecid	1	90.3 ± 7.9	90.2 ± 10
+Estron-3 sulphate	1	93.0 ± 4.6	94.9 ± 5.6
+PAH	1	83.1 ± 5.3	107 ± 9

[^3^H]L-Carnitine uptake was evaluated for 15 min in the presence or absence of various transporter inhibitors and substrates at a concentration of 0.5–1 mM in NSC-34 cell lines at 37 °C and pH 7.4. The presented values represent the mean ± SEM (*n* = 3–4). *** *p* < 0.001 and ** *p* < 0.01 denote levels of significant difference from their respective controls. ALC: acetyl L-carnitine TEA: tetraethyl ammonium, MPP^+^1: methyl-4-phenylpyridinium ion, GABA: gamma-aminobutyric acid, PAH: para-aminohippuric acid.

## Data Availability

This study did not report any data.
